# *Hibiscus acetosella*: An Unconventional Alternative Edible Flower Rich in Bioactive Compounds

**DOI:** 10.3390/molecules28124819

**Published:** 2023-06-16

**Authors:** Laila Yasmim dos Santos Silva, Andrezza da Silva Ramos, Débora Nogueira Cavalcante, Valdely Ferreira Kinupp, Jojo Silva Rodrigues, Bianca Muniz Lacerda Ventura, Tiago Antônio de Oliveira Mendes, Edgar Aparecido Sanches, Pedro Henrique Campelo, Jaqueline de Araújo Bezerra

**Affiliations:** 1 Analytical Center, Campus Manaus Center, Science and Technology of Amazonas, Manaus 69020-120, Brazil; 2019005776@ifam.edu.br (L.Y.d.S.S.); andrezza.ds.ramos@gmail.com (A.d.S.R.); 2018000668@ifam.edu.br (D.N.C.); 2 Federal Institute of Education, Science and Technology of Amazonas, Manaus 69020-120, Brazil; valdely.kinupp@ifam.edu.br; 3 Department of Biochemistry and Molecular Biology, Federal University of Viçosa, Viçosa 36570-000, Brazil; joao.rodrigues5@ufv.br (J.S.R.); bianca.ventura@ufv.br (B.M.L.V.); tiagoaomendes@ufv.br (T.A.d.O.M.); 4 Laboratory of Nanostructured Polymers, Materials Physics Department, Federal University of Amazonas, Manaus 69067-005, Brazil; sanchesufam@ufam.edu.br; 5 Department of Food Technology, Federal University of Viçosa, Viçosa 36570-000, Brazil; pedrocampelo@ufv.br

**Keywords:** vinagreira-roxa, bioactive compounds, antioxidant, delphinidin, myricetin

## Abstract

The interest in the consumption of edible flowers has increased since they represent a rich source of bioactive compounds, which are significantly beneficial to human health. The objective of this research was to access the bioactive compounds and antioxidant and cytotoxic properties of unconventional alternative edible flowers of *Hibiscus acetosella* Welw. Ex Hiern. The edible flowers presented pH value of 2.8 ± 0.00, soluble solids content of 3.4 ± 0.0 °Brix, high moisture content of about 91.8 ± 0.3%, carbohydrates (6.9 ± 1.2%), lipids (0.90 ± 0.17%), ashes (0.4 ± 0.0%), and not detectable protein. The evaluation of the scavenging activity of free radicals, such as 2,2-diphenyl-1-picryl-hydrazyl (DPPH) and 2,2′-azinobis-(3-ethylbenzothiazoline-6-sulfonic acid) (ABTS), of the flower extract was better than the results observed for other edible flowers (507.8 ± 2.7 μM TE and 783.9 ± 30.8 μM TE, respectively) as well as the total phenolic composition (TPC) value (568.8 ± 0.8 mg GAE/g). These flowers are rich in organic acids and phenolic compounds, mainly myricetin, and quercetin derivatives, kaempferol, and anthocyanins. The extract showed no cytotoxicity for the cell lineages used, suggesting that the extract has no directly harmful effects to cells. The important bioactive compound identified in this study makes this flower especially relevant in the healthy food area due to its nutraceutical potential without showing cytotoxicity.

## 1. Introduction

The growing interest in edible flowers is also due their commercial value and to their intrinsic characteristics, such as aroma, exotic textures, delicate flavor, attractive color, but mainly to their chemical composition rich in anthocyanins, flavonoids, and phenolic acids reported as beneficial to human health [1] and that justifies the biological properties that edible flowers have [2,3].

The edible flowers have been consumed as food in several cultures worldwide as part of traditional cuisine or alternative medicine [2,4]. They may contain several natural constituents with antioxidant potential. Studies with more common species, such as calendula, rose, hibiscus, jasmine, or lavender flowers, pointed to their antioxidant potential related to the polyphenol content [5,6]. However, there is a shortage of information about its antioxidant and nutraceutical potential. Information on the derivatives of flavonol and anthocyanin in edible flowers of the genus *Hibiscus* is still limited, and their chemical compositions indicate the importance of further studies on the antioxidant and nutritional potential of *Hibiscus* flowers.

*Hibiscus* species are native to tropical and subtropical regions, producing antioxidant phenolic compounds and flavonoids in plant tissues as protection against oxidative damage derived from exposure to ultraviolet light [7]. Some reports have indicated that the genus *Hibiscus* contains about 275 species of flowering plants widely cultivated in the tropics and subtropics, as well revealing excellent anticancer activity in the lungs, breast, and liver [8]. Roselle (*H. sabdariffa*) is reported to prevent obesity-related insulin resistance [9], which can progress to a number of metabolic disorders.

The most studied species from the genus *Hibiscus* is *H. sabdariffa* L., known as hibisco. The flower’s calyx is considered a food rich in lutein, chlorogenic acids, and anthocyanins, mainly delphinidin 3-*O*-sambubioside. The *Hibiscus* extract is effective in breast cancer and may complement chemotherapy regimens as an adjuvant to reduce chemotherapy dosages and toxicity. This species has been widely used in traditional medicine due to its high content of polyphenols. In addition, there is an important perspective on its therapeutic uses due to the presence of a bioactive that acts in the prevention of chronic and degenerative diseases associated with oxidative stress [10].

The *Hibiscus acetosella* Welw. Ex Hiern species is a member of the Malvaceae family, native to Africa and commonly consumed as a green vegetable. In traditional medicine in West and Central Africa, decoction drinks are prepared from leaf and bud extracts due to their anti-anemic and antipyretic properties. In Brazil, *H. acetosella* is known as “vinagreira-roxa”, “vinagreira”, “groselheira (gooseberry bush)”, “rosela”, “quiabo azedo (sour okra)”, and “quiabo roxo (purple okra)”. It is a sub-woody shrub presenting 1.5 to 3.0 m in height and is considered a non-conventional food plant (known in Brazil as “PANC”) with simple leaves that vary from green to completely purple and has solitary flowers with purple petals. It is cultivated throughout the country for ornamental purposes, and its leaves are consumed *in natura*; however, its flowers have been also consumed in salads and gourmet dishes [11].

The main objective of this study was to determine the centesimal composition, physicochemical properties, bioactive compounds (polyphenols and phenolic acid), health-promoting properties (antioxidant), and cytotoxicity of edible flowers of *H. acetosella* Welw. Ex Hiern. These unpublished data can significantly contribute to new directions on the use of edible flowers presenting pro-health properties, as well as contribute with significant knowledge to the scientific community on the chemistry of the edible flowers of the purple vinegar tree.

## 2. Results and Discussion

### 2.1. Physicochemical Characterization, Centesimal Composition, and Antioxidant Capacity

The physicochemical properties of the *H. acetosella* flowers are important parameters, especially because there is a lack of information on this species. The obtained results are shown in Table 1. Part of the results of centesimal composition and physicochemical properties revealed high moisture (91.76 ± 0.27%) and low ash (0.45 ± 0.01%) contents. The °Brix values were measured from the soluble solids content of the solution. A 100 g sample solution measuring 50 °Brix has 50 g of sugar and other dissolved solids in addition to 50 g of water. Sugars are the most abundant soluble solid in fruit and vegetable juices. The °Brix values can be useful in the variety selection, harvest scheduling, flavor, or sweetness [12].

The observed values are usually found in edible flowers of different genus and even in *Hibiscus*. Several reports on studies of edible flowers that have been used as food ingredients in Japan from the point of view of nutraceutical ingredients, report similar content of proteins, saccharides, and fats [13]. The authors also highlighted that edible flowers are a potential source of antioxidants, and their addition to the human diet can bring several health benefits.

The *Hibiscus* flower *H*. *sabdariffa* L. has been used in traditional Chinese medicine in the form of tea to treat hypertension and inflammation [14]. Studies have revealed that *H*. *cannabinus*, in addition to presenting food potential, has a wide range of therapeutic properties, including antioxidant, antimicrobial, anticancer, antihyperlipidemic, antiulcerogenic, anti-inflammatory, and hepatoprotective activities [15,16]. The consumption of *H. manihot* L. flowers tea present a positive response to vasodilating activity, which is related to the presence of flavonoids [17]. The tea from edible *T. speciosum* flowers presented protocatechuic acid, quercetin, quercetin pentoside, and quercetin-3-*O*-glucoside [18]. Polyphenols are natural compounds found abundantly in vegetables and fruits, and they play a significant role in many physiological and metabolic processes [19], such as reducing the risk of neurodegenerative diseases, cancer, diabetes, and metabolic disorders associated with obesity [20]. The results from the DPPH and ABTS assays as well as the TPC quantification in the extract of *H*. *acetosella* flowers are shown in Table 1.

The extract of *H*. *acetosella* flowers showed TPC content within a range of expected values for edible flowers of the Malvaceae family. The *T. speciosum* extract presented a slight higher TPC value (640 mg EAG/g) [18], and *H*. *rosa-sinensis* L. showed a lower value of antioxidant activity (DPPH 145 ± 3 mg TE/100 g DW) [21] than the extract of *H*. *acetosella* flowers. Several studies both in vitro and in vivo have shown that extracts of *Hibiscus* present a potent antioxidant effect due to its strong scavenging effect on reactive oxygen and free radicals [19,22]. The results found herein contribute to stimulating the consumption of edible flowers as a functional food as well as stimulating their use as sources of natural antioxidants by the food industry [23].

### 2.2. Analysis of Hibiscus acetosella Flowers Extract by ^1^H NMR

**Figure 1:**
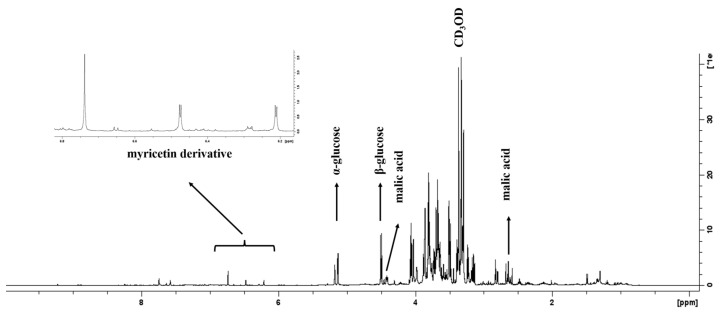
^1^H NMR spectrum of the hydroethanolic extract of *H. acetosella* flowers (CD_3_OD, 11.74T) highlighting the region from 5.8 to 6.8 ppm.

The characteristic signals of carbohydrates, organic acids, and aromatic compounds were observed and are shown in Figure 1. Signals δH at 5.13 ppm (d, *J* = 3.7 Hz) and 4.50 ppm (d, *J* = 7.8 Hz) were assigned to α-glucose and β-glucose, respectively. The signal δH at 1.49 ppm (d, *J* = 7.2 Hz) is related to alanine. The signals δH at 4.42 ppm (dd, *J* = 7.3:4.3 Hz), 2.63 ppm (dd, *J* = 16.1:7.3 Hz), and 2.81 ppm (dd, *J* = 16.3:4.3 Hz) were attributed to malic acid. Several signals were found in the characteristic region of aromatic compounds, and the highest intensity is characteristic of a flavonoid skeleton with a singlet at δH 6.74 ppm referring to the B ring as well as a pair of doublets at δH 6.47 (d, *J* = 2.0 Hz) and δH 6.21 (d, *J* = 2.0 Hz) related to the hydrogens of the A ring. Based on the flavonoid profile and chemical shifts, a myricetin derivative is suggested, which is a substance previously reported in *Hibiscus* species [24]. The edible flowers of the *Hibiscus* species are rich in bioactive secondary metabolites [21]. In general, the skeletal structures of anthocyanins detected in the flowers of *H. syriacus* were cyanidin, delphinidin, procyanidin, peonidin, pelargonidin, petunidin, malvidin, and dihydroflavonoids [25].

### 2.3. Determination of Bioactive Compounds by HRMS and HPLC-DAD

The compounds identified by high resolution mass spectrometry (HRMS) in the hydroethanolic extract of *H. acetosella* flowers are described in Table 2 and quantified by HPLC-DAD (Table 3 and Table 4). The compounds were previously described in other species of *Hibiscus* [24,26]. The molecule as oxalosuccinic acid was already identified in *rosa-sinensis* L. red flowers [27]. The flowers of *H. rosa-sinensis* are known in the local medicines of India and China for their antipyretic, analgesic, anti-asthmatic, and anti-inflammatory properties in addition to their flavoring potential for beverages [28]. It is possible to find flavonoids, tannins, alkaloids, steroids, terpenoids, amino acids, and glycosides in the ethanolic and aqueous extracts of the flowers of *H. rosa-sinensis* [29].

Gallic acid 3-*O*-β-glucoside was related in Malvaceae [30]. Another phenolic identified as 5-(3-carboxy-2,5-dihydroxyphenyl)-2,4-dihydroxy-3-methoxybenzoic acid was identified in *Hibiscus* spp. [31]. A wide variety of bioactive substances, such as polyphenols, flavonoids, and anthocyanins, have been reported in the *Hibiscus* species. Two species stand out, *H*. *cannabinus* and *H*. *sabdariffa*, which have been extensively studied due to the relationship between their biochemical compounds and biofunctional activity, while few reports have been found on *H*. *acetosella* [32]. Different bioactive compounds found in this study can contribute to revealing their nutraceutical and pharmaceutical potential, as already reported in in vitro and in vivo studies. Organic acids and phenolic compounds were identified, mainly the flavonoid derivatives of myricetin, quercetin, and kaempferol and the anthocyanins of delphinidin and cyanidin. Intaking of flavonoid-rich foods and beverages lowers the risk of chronic disease and mortality in supervision studies, as demonstrated by converging evidence from in vitro and clinical studies [33,34]. 

The results show great variability of bioactive compounds in *H*. *acetosella* edible flowers. Comparing our data with those reported in literature, the bio-accessibility evaluated of phenolic compounds from eight edible flowers, namely mini-rose, torenia, mini-daisy, clitoria, cosmos, cravine, begonia, and marigold, through an in vitro digestion system presented phenolic acids, stilbenes, flavanol, anthocyanin, flavonol, and flavanone in their compositions in different proportions in each flower, and some of them with greater bioavailability of phenolic compounds presenting significant antioxidant activity, such as cosmos and mini-rose [3]. 

**Figure 2:**
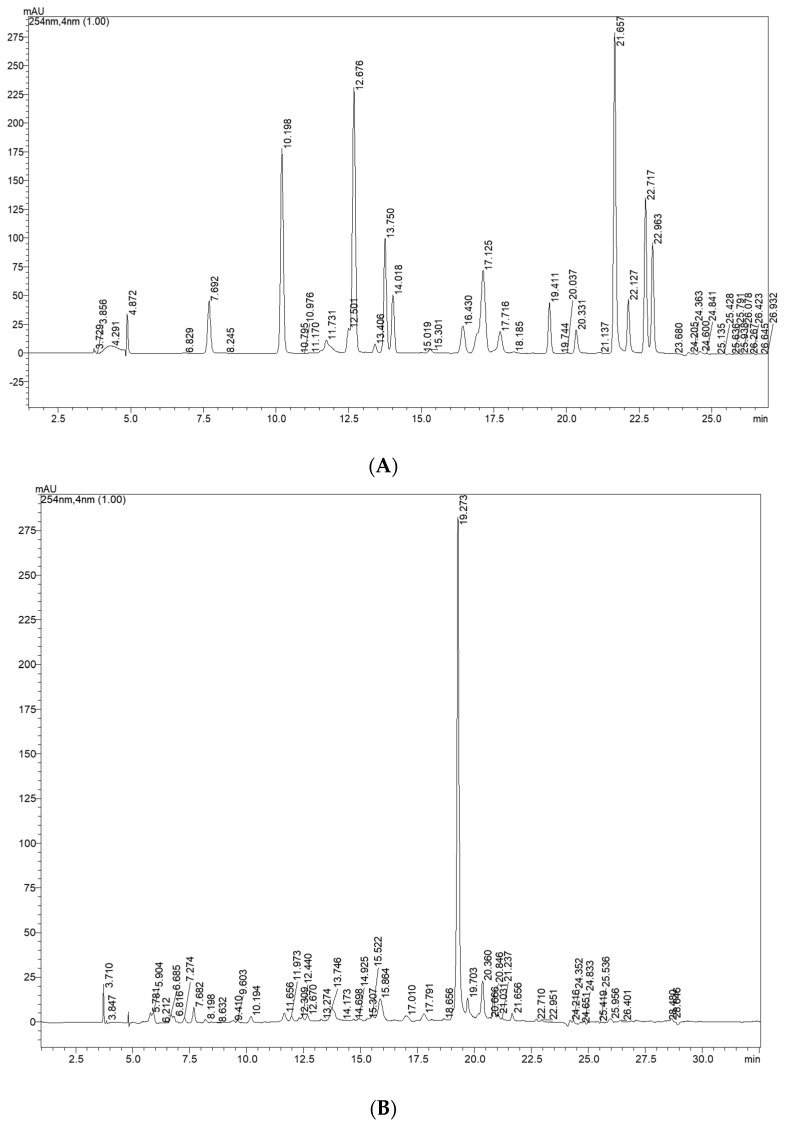

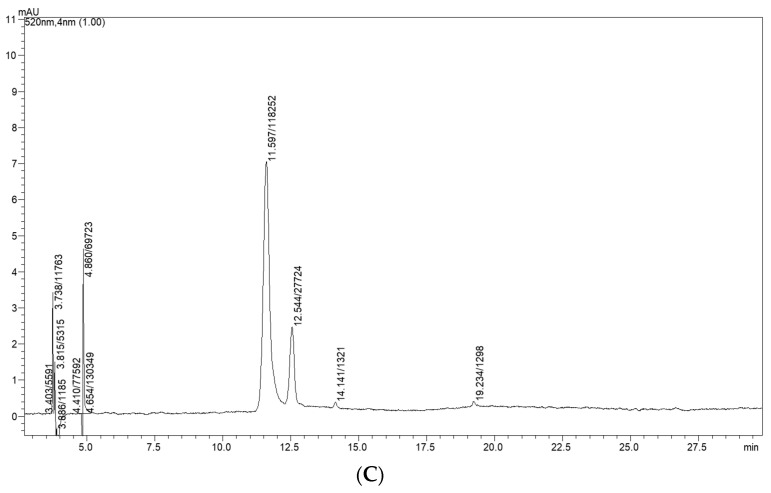
Chromatograms obtained by HPLC-DAD: standard detection at 254 nm (**A**); extract of *H. acetosella* detected at 254 nm (**B**) and at 520 nm for anthocyanins (**C**).

Table 3 and Table 4 show the main bioactive compounds quantified using the external standard method. The sequence is according to its retention time observed in chromatogram (Figure 2). The bioactive compounds, flavonol and anthocyanin derivatives, were identified by matching DAD features with those available in literature and quantified by response factor based on chemical structure of analytical standard; flavonoids derivatives are the major constituents, followed by cinnamic acid derivatives. In Figure 2C it is possible to observe the maximum band absorption of anthocyanin at 520 nm. An intense signal in 19.27 min retention time (Figure 2A) is confirmed through the UV-band maximum absorption characteristic of the flavonol derivative. That was quantified with the relative response factor using an analytical standard of flavonol. The amounts of gallic, protocatechuic acid, and quercetin derivatives, powerful antioxidants, were higher than the values determined in *Hibiscus* flowers (*Hibiscus rosa-sinensis* L.) [21].

The flowers of *H. syriacus* are consumed mainly by their delicate and colorful flowers that vary among white, pink and purple. According to previous reports, the flowering period extends from May to October, but the individual flowers stage is relatively short, lasting only one day. This can cause some difficulties in collecting and storing fresh flowers; all strains of *H. syriacus* were collected in the city of Senshan, Yiwu Zhejiang province, China, on 14 August 2019 for the analyses. The study highlights that the stability of anthocyanins in *H. syriacus* flowers is easily affected by environmental and chemical factors, such as light, pH, ascorbic acid, H_2_O_2_, and Na_2_SO_3_. However, the collection period is not mentioned as an interfering factor for the stability of anthocyanins. In the methanolic extracts of three strains of *H. syriacus*, the total anthocyanin contents were analyzed by the pH differential method. As a result, the red flowers of *H. syriacus* accumulated more anthocyanins than the purple and white flowers, with values of 3.2 mg/g, 1.87 mg/g, and 1.61 mg/g, respectively [25]. This preliminary study associating the color variations of *H. syriacus* flowers with their chemical compositions may help to understand the anthocyanin value found in the reddish flowers of *H. acetosella* (approximately 3.18 mg/g of flowers). These values are similar with that presented in this study to flowers of *H. acetosella* whose petals are light red with a dark red. In hibiscus flowers, the most abundant chemical class is flavonols, and several flavonols were identified and described for the first time in *H. acetosella* flowers in this work.

### 2.4. Cytotoxicity Evaluation

**Figure 3:**
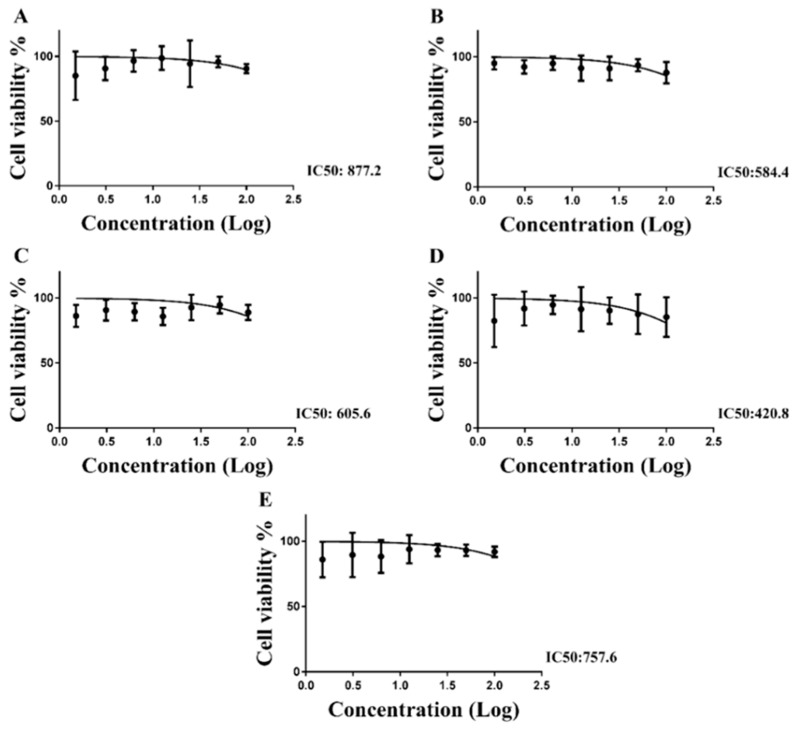
Cytotoxicity profile of extract. The cytotoxicity was determined via MTT (3-(4,5-dimethyl-thiazol-2-yl)-2,5-diphenyltetrazolium bromide) colorimetric assay. Cell lineages: Vero cells (**A**), HEPG2 cell (**B**), HEK cells (**C**), RAW cells (**D**), L6 cells (**E**). The CC_50_ values (cytotoxic concentration that decreases cell viability by 50%) correspond to the average of three independent experiments.

The extract exhibited no cytotoxicity effects on the cell lineages used (different 2D-cell cultures were used to measure in vitro renal, embryonic, hepatic, cardiac, and blood), even in the maxima concentration used (Figure 3). The result suggested that the extract is safe for the cells with no directly harmful effects to cells. Previous studies on *Hibiscus* spp. flowers have already proven the non-toxicity of the flowers as well as the isolated chemical constituents from these flowers, which makes them edible [35,36]. There are several studies on the cytotoxicity of leaves and other parts of the species *H. acetosella*, but no reports on the toxicity of its flowers have been found in scientific literature.

## 3. Materials and Methods

### 3.1. Collection and Processing Flower Sample

The flowers of *H. acetosella* were collected at the PANC site located at the Estrada do Brasileirinho, 4960, Km 6, Industrial District II, Manaus–AM (3°6′26″ S/60°1′34″ W, SISGEN authorization A26CD5E). Flowers were analyzed in fresh form as well as subjected to an ethanol-water solution (8:2) extraction in an ultrasonic bath for 15 min in triplicate.

### 3.2. Chemical Physical Analysis and Centesimal Composition

The pH was determined using 5 mL of flower juice using a pH meter (AKSO–AK90) previously calibrated [37]. The measurement of total soluble solids was determined with a digital refractometer (HI 96801, Hanna Instruments, Woonsocket, RI, USA) using 3 drops of the sample; the results are expressed in °Brix. All measurements were performed in triplicate.

Flower moisture was obtained by heating in an oven at 105 °C for 3 h. The heating and cooling operation was repeated until constant weight. The ash content was measured by carbonization followed by incineration of the flowers in a muffle at 550 °C. Determination of protein content by the classic Kjeldahl method. 0.2 g of the samples were weighed and transferred to a Kjeldahl tube, and 5 mL of sulfuric acid and 1.2 g of catalytic mixture (contains copper sulfate, titanium oxide and potassium sulfate) were added. The digestion process was carried out and after the displacement of the nitrogen present in the sample, the nitrogen distillation process was carried out, using 2 drops of phenolphthalein and 0.2 g of zinc powder; the flask was connected to the distillation system of nitrogen. The excess of the 0.05 M sulfuric acid was titrated with 0.1 M sodium hydroxide solution until obtaining 50 mL of distillate. This analysis was performed in triplicate. Protein is calculated using the following Equation (1):

(1) $$\%\mathrm{protein}\left(m/m\right)=\frac{V\times 0.14\times f}{W}$$

where V = difference between the volume (mL) of sulfuric acid and sodium hydroxide spent in the titration; W = weight (g) of the sample; f = conversion factor (6.25).

The extraction of total lipids was performed with 2 g of the sample using the Soxhlet method with hexane in continuous flow for 8 h [37]. All measurements were performed in triplicate.

### 3.3. DPPH^•^ and ABTS^•+^ Radicals Scavenging Capacity Assay

For the DPPH^•^ (2,2-diphenyl-1-picryl-hydrazyl) radical assay, 100 μL of the sample was added to 3.9 mL of DPPH^•^ solution (100 μM) and incubated in the dark for 30 min [38]. The absorbance was measured at 515 nm. The scavenging capacity of radical cations ABTS^•+^ [2,2′-azinobis-(3-ethylbenzothiazoline-6-sulfonic acid)] was performed with a volume of 3.0 mL of the obtained solution (7 mM ABTS) mixed with 30 μL of *H. acetosella* extract and was allowed to react in the dark for 6 min. The absorbance was measured at 734 nm [39]. The Trolox standard calibration curve was constructed at different concentrations (250 to 2000 μM). These assays were performed in triplicate, and the results are expressed in micromolar of Trolox equivalent (μM TE). The assay measure was made with an ultraviolet-visible spectrophotometer (nova NI 2200, Nova Instruments, Campinas, Brazil).

### 3.4. Total Phenolic Composition

An aliquot of 200 μL (1 mg/mL) of the hydro-ethanolic extract of flowers was reacted with 1.5 mL of Folin Ciocalteu reagent/water (1:10) for 5 min. Then, 1.5 mL of sodium bicarbonate (60 g/L) were added to this previous solution. After 90 min of reaction in the dark, the absorbance measurements were obtained using an ultraviolet-visible spectrophotometer at 725 nm (nova NI 2200, Nova Instruments, Campinas, Brazil). The standard curve of gallic acid was constructed at different concentrations (31.2 to 1000 μg/mL). This assay was performed in triplicate, and the results were expressed in milligrams of gallic acid equivalent per gram (mg GAE/g) [40].

### 3.5. Bioactive Compounds Identification

#### 3.5.1. Chemical Profile by Nuclear Magnetic Resonance

The chemical profile of the flowers extract was obtained on an ^1^H 500 MHz NMR Spectrometer Bruker^®^, BBFO Plus SmartProbeTM (New York, NY, USA). The extract was solubilized using deuterated methanol (CD_3_OD) and transferred to a 5 mm ^1^H NMR tube [41]. The spectrum was processed using the software Topspin 4.1.1 (Bruker, Karlsruhe, Germany).

#### 3.5.2. Identification by High Resolution Mass Spectrometry (HRMS)

The HRMS analysis was performed on an ESI-MicroTOF-Q II hybrid quadrupole time-of-flight mass spectrometer (Bruker Daltonics^®^, Fremont, CA, USA). The sample (1 mg/mL) was diluted in methanol/water (1:1, *v*/*v*) with 0.1% formic acid and 3 mM ammonium formate. The mass spectrometer parameters were as follows: capillary voltage (−3.5 kV for negative and 4.5 kV for positive ion modes); nebulizer gas (nitrogen, 2.0 bar); dry gas (nitrogen, 6.0 L/min); and mass range (*m/z* = 100–800 Da) [26]. The instrument was calibrated with sodium formate. Data acquisition and processing were performed using the software Bruker^®^ Compass Data Analysis 4.1.

### 3.6. Quantification of Bioactive Compounds by HPLC-DAD

The HPLC analyses were conducted using a Shimadzu Shim-pack octadecyl silane (ODS) column (ID 5 µm, 250 × 4.6 mm) equipped with a precolumn of the same material; the oven temperature was maintained at 30 °C. The mobile phase was water (A) and methanol (B); the acidity was obtained using phosphoric acid at pH 3, gradient from 1.0 min in isocratic mode at 10% (B), 10–50% (B) in 13 min, 50–70% (B) in 6 min, 70–100% (B) in 7 min, followed by elution with 100% methanol for 5 min. The chemical profile and quantification were obtained on a Shimadzu Prominence LC-20AT (Shimadzu Corporation Co., Ltd., Kyoto, Japan), equipped with a DGU-20A5 degasser equipped with SPD-M20A (PDA) detector. The linearity was evaluated by analysis of external standard stock solution from 0.5 to 0.0156 mg/mL in triplicate. The equation parameters (slope and intercept) of each standard curve were used to obtain the samples concentrations. The limits of detection (LOD) and quantitation (LOQ) were calculated from a calibration curve by dividing the standard deviation of the calibration curve by its slope multiplied by 3.3 and 10.0, respectively [42].

#### Quantification by Relative Response Factor

Quantitative analysis of flavonol and cinnamic acid derivatives was performed by establishing response factors from caffeic acid, sinnapic acid, myricetin, and quercetin standard selected as reference [43]. The response factors (RF) for the flavonol and cinnamic derivatives were calculated as a ratio of the concentration in relation to the corresponding area of standard sample [44]. The relative response factors (RRF) were calculated as the ratio of the RF for each analyte to that of the chosen reference. The quantification of phenolic derivatives content in the sample was carried out according to the following Equation (2):

(2) $$\mathrm{Content}\;\left(\%,\frac{w}{w}\right)=\frac{A_{\mathrm{samp}}\times \mathrm{RRF}\times R_{f}\times V_{\mathrm{samp}}\times 100}{W_{\mathrm{samp}}\times 1000}$$

where A_samp_: area due to the phenolic in the sample (mAU·s); RRF: the average relative response factor of that phenolic derivative to the reference phenolic; R_f_: response factor of the phenolic standard [(μg/mL)/mAU·s]; V_samp_: volume of sample solution (mL); W_samp_: sample weight (μg).

### 3.7. Cytotoxicity Evaluation

In order to evaluate the toxicity of extract, the colorimetric assay of MTT (3-(4,5-dimethyl-thiazol-2-yl)-2,5-diphenyltetrazolium bromide) was used to determine the cytotoxic concentration that decreases cell viability by 50% (CC_50_) from cell cultures of African green monkey kidney epithelial Vero (ATCC^®^ CCL-81™), liver epithelial-like HEPG2 (ATCC^®^ HB-8065™), human kidney embryo HEK-293 (ATCC^®^ CRL-1573™), mouse macrophage RAW 264.7 (ATCC^®^ TIB-71™), and rat myoblast L6 (ATCC^®^ CRL-1458™) [45]. A suspension of each cell type, containing approximately 1 × 10^4^ cells/mL, was seeded in a 96-well plate (100 µL/well) until reaching 70–80% confluence. After 24 h or until reaching confluence, the cells were treated with a different serial concentration of the extract in dilution factor of 10 in incomplete DMEM (Dulbecco’s Modified Eagle’s Medium) medium, starting with a concentration of 100 µg/mL. The dilutions were incubated for 24 h at 37 °C under a 5% CO_2_ atmosphere. Subsequently, the medium was then removed, and 100 µL of MTT (0.5 mg/mL) was added, followed by a new incubation period using the same conditions described above. Then, the MTT medium was removed and replaced with 100 µL of DMSO (dimethyl sulfoxide) per well to dissolve the formazan crystals. The plate was then shaken for 20 min, and the reading was performed in a spectrophotometer at 540 nm.

### 3.8. Statistical Analysis

The cytotoxicity results (CC_50_) were obtained through non-linear regression analysis of the percentages of inhibition of cell viability related to different concentrations of compounds using the software GraphPad Prism version 6.0 [46]. The CC_50_ values represented the average of three independent experiments.

The results of the scavenging of DPPH^•^/ABTS^•+^ radicals and total phenolic content were expressed as mean ± standard deviation and were evaluated using Microsoft Excel^®^ 2016. Statistical significance was determined using ANOVA (one-way) followed by multiple comparison using the Tukey test (95% confidence level). Values of *p* ≤ 0.05 were considered significant.

## 4. Conclusions

This research contributes scientifically to the chemical knowledge of the edible flowers of *H. acetosella*, while also highlighting the value of the selected species. The chemical composition is similar to other species of this genus. The flower extract is rich in bioactive compounds, including myricetin, gallic acid, delphinidin 3-*O*-glucoside, and caffeic acid, which contribute to its antioxidant properties. The chemical profile reveals the presence of flavonoid derivatives as well as other compounds, such as organic acids. This study is the first to report data on the cytotoxic activity of the flowers of this species. The occurrence of *H. acetosella* flowers in Brazil allows for their consumption in various food applications. For this reason, our results encourage further studies to assess the effectiveness and safety of consuming this species for the benefit of human health.

## Figures and Tables

**Table 1:** Physicochemical characterization, centesimal composition, antioxidant capacity, and total phenolics content (TPC) of *H. acetosella* flowers.

Parameters	Mean ± SD
pH	2.8 ± 0.0
Soluble solids (°Brix)	3.4 ± 0.0
Moisture (g/100 g)	91.76 ± 0.27
Ashes (g/100 g)	0.45 ± 0.01
Lipids (g/100 g)	0.90 ± 0.17
Protein (g/100 g)	ND
Carbohydrates (g/100 g)	6.89 ± 0.19
Calories (kcal)	34.7 ± 0.45
DPPH (μM TE)	507.8 ± 2.7 ^c^
ABTS (μM TE)	783.9 ± 30.8 ^a^
TPC (mg GAE/g)	568.8 ± 0.8 ^b^

Data are the mean of = 3 determinations ± SD (mean standard deviation). Means that do not share the same letter are significantly different.

**Table 2:** Bioactive compounds identified by HRMS in *H. acetosella* flower extract.

**Compound**	**Molecular Ion**	**[M − H]^−^ *m/z***	**[M − H]^−^ *m/z*** **(Error in ppm)**
Mallic acid	C_4_H_5_O_5_	133.0142	133.0142 (0.5)
Oxalosuccinic acid	C_6_H_5_O_7_	189.0041	189.0043 (−1.0)
Quercetin	C_15_H_9_O_7_	301.0354	301.0352 (0.7)
Myricetin	C_15_H_9_O_8_	317.0303	317.0295 (2.3)
Gallic acid 3-*O*-β-glucoside	C_13_H_15_O_10_	331.0671	331.0640 (9.4)
5-(3-Carboxy-2,5-dihydroxyphenyl)-2,4-dihydroxy-3-methoxybenzoic acid	C_15_H_11_O_9_	335.0409	335.0410 (−0.3)
Caffeoyl-hydroxycitric acid	C_15_H_13_O_11_	369.0463	369.0476 (−3.4)
Quercetin 3-*O*-rhamnoside (quercitrin)	C_21_H_19_O_11_	447.0933	447.0930 (0.5)
Quercetin 3-*O*-glucoside	C_21_H_19_O_12_	463.0882	463.0863 (4.1)
Myricetin 3-*O*-glucoside	C_21_H_19_O_13_	479.0831	479.0833 (0.3)
3,5-di-*O*-galloylquinic acid	C_21_H_19_O_14_	495.0780	495.0788 (1.6)
Quercetin 3-*O*-β-D-(1″-*O*-malonyl)-xylopyranoside	C_23_H_17_O_14_	517.0624	517.0627 (0.7)
Quercetin 3-*O*-(6′-*O*-malonyl)galactoside	C_24_H_19_O_15_	547.0729	547.0731 (0.3)
Delphinidin 3-*O*-(6″-*O*-malonyl)-β-glucoside	C_24_H_21_O_15_	549.0886	549.0896 (−1.8)
Kaempferol-3-*O*-sambubioside	C_26_H_27_O_15_	579.1355	579.1351 (−0.7)
Quercetin-3-*O*-sambubioside	C_26_H_27_O_16_	595.1305	595.1298 (1.1)
Cyanidin 3-*O*-β-D-caffeoylglucoside	C_30_H_25_O_14_	609.1250	609.1258 (−1.3)
Myricetin-3-arabinogalactoside	C_26_H_27_O_17_	611.1254	611.1264 (−1.7)
Miricetin 3-*O*-β-D-glucosil-(1→2)-β-D-glucoside	C_27_H_29_O_18_	641.1359	641.1388 (4.4)
**Compound**	**Molecular Ion**	**[M + H]^+^ *m/z***	**[M + H]^+^ *m/z*** **(error in ppm)**
Delphinidin	C_15_H_11_O_7_	303.0499	303.0409 (−3.1)
Cyanidin 3-*O*-β-D-glucoside	C_21_H_21_O_11_	449.1078	449.1088 (−2.1)
Delphinidin 3-glucoside	C_21_H_21_O_12_	465.1028	465.1035 (−1.6)
Cyanidin 3-sambubioside	C_26_H_29_O_15_	581.1501	581.1495 (1.1)
Delphinidin 3-sambubioside	C_26_H_29_O_16_	597.1450	597.1453 (0.5)
Delphinidin 3,5-*O*-diglucoside	C_27_H_31_O_17_	627.1556	627.1558 (0.4)

**Table 3:** Retention time (RT), maximum absorbance (λmax), and validation parameters of the HPLC-DAD methodology for the determination of the target bioactive compounds in *H. acetosella* edible flowers.

RT (min)	Bioactive Compound	λmax (nm)	Conc. (μg/mL)	SD *	RSD% ^ǂ^	R	R^2^	Calibration Curve
7.68	Gallic acid	271	322	0.06	19.91	0.998	0.996	y = 0.75 × 10^6^ X + 12,790.7
10.19	Protocatechuic acid	293	44	0.01	18.64	0.998	0.996	y = 2.85 × 10^6^ X + 50,231.1
11.62	Cyanidin 3-*O*-glucoside	279/529	201	0.09	42.33	0.968	0.937	y = 0.44 × 10^6^ X − 11,172
12.54	Cyanidin	519	10	0.00	19.85	0.993	0.987	y = 3.88 × 10^6^ X + 85,884
13.20	Delphinidin 3-*O*-glucoside	521	243	0.43	23.84	0.994	0.988	y = 0.10 × 10^6^ X + 3339.92
13.76	Caffeic acid	324	237	0.04	18.08	0.993	0.987	y = 1.57 × 10^6^ X + 26,680
17.00	Sinapic acid	324	65	0.01	17.79	0.998	0.996	y = 2.19 × 10^6^ X 36,087.4
20.32	Myricetin	256/375	363	0.15	25.24	0.996	0.992	y = 0.05 × 10^6^ X − 9668.84
21.63	Quercitrin	254/371	65	0.00	15.93	0.998	0.997	y = 4.02 × 10^6^ X + 54,454.1
22.12	Luteolin	255/349	13	0.00	16.36	0.999	0.999	y = 0.83 × 10^6^ X + 5952.23

* = standard deviation; ^ǂ^ = relative standard deviation.

**Table 4:** Bioactive compounds content (%, *w*/*w*, as internal standard) quantified by relative response factor (RF).

RT (min)	Bioactive Compound	λmax (nm)	* RF	** Content	*** LOQ	**** LOD
	Anthocyanidin standard		1.92974 × 10^−6^		−1.73 × 10^−9^	−5.70 × 10^−9^
11.95	Anthocyanin derivative	277/529	9.90959 × 10^−7^	0.080		
	Cinnamic standard		837,181.4346		6.03 × 10	1.99 × 10
12.70	Cinnamic acid	326	72,848.10127	0.021		
14.67	Cinnamic derivative	326	32,050.63291	0.009		
	Flavonol standard		20,175.78773		4.27 × 10	1.41 × 10
15.27	Flavonol rutinoside derivative	355	184,461.5385	0.070		
15.48	Flavonol rutinoside derivative	346	267,748.7179	0.102		
18.11	Flavonol derivative	369	117,369.146	0.082		
19.27	Flavonol derivative	254/370	1.28163 × 10^−7^	5.46		
19.70	Flavonol derivative	252/370	154,988.9807	0.108		
	Flavone standard		518,203.8567		9.28 × 10	3.06 × 10
20.81	Flavone derivative	369	3870.52091	0.002		

* (mAU/μg mL^−1^); ** (%, *w*/*w*); *** = limit of quantification (10 × SD/b); **** = limit of detection (3.3 × SD/b).

## Data Availability

Not applicable.

## References

[1] Nowicka P., Wojdyło A. (2019). Anti-Hyperglycemic and Anticholinergic Effects of Natural Antioxidant Contents in Edible Flowers. Antioxidants.

[2] Takahashi J.A., Rezende F.A.G.G., Moura M.A.F., Dominguete L.C.B., Sande D. (2019). Edible flowers: Bioactive profile and its potential to be used in food development. Food Res. Int..

[3] De Morais J.S., Sant’Ana A.S., Dantas A.M., Silva B.S., Lima M.S., Borges G.C., Magnani M. (2020). Antioxidant activity and bioaccessibility of phenolic compounds in white, red, blue, purple, yellow and orange edible flowers through a simulated intestinal barrier. Food Res. Int..

[4] Dos Santos I.C., Reis S.N. (2021). Edible flowers: Traditional and current use. Ornam. Hortic..

[5] Fernandes L., Casal S., Pereira J.A., Saraiva J.A., Ramalhosa E. (2017). Edible flowers: A review of the nutritional, antioxidant, antimicrobial properties and effects on human health. J. Food Compos. Anal..

[6] Purohit S.R., Rana S.S., Idrishi R., Sharma V., Ghosh P. (2021). A review on nutritional, bioactive, toxicological properties and preservation of edible flowers. Futur. Foods.

[7] Zhen J., Villani T.S., Guo Y., Qi Y., Chin K., Pan M.-H., Ho C.-T., Simon J.E., Wu Q. (2015). Phytochemistry, antioxidant capacity, total phenolic content and anti-inflammatory activity of *Hibiscus sabdariffa* leaves. Food Chem..

[8] Alam P., Al-Yousef H.M., Siddiqui N.A., Alhowiriny T.A., Alqasoumi S.I., Amina M., Hassan W.H.B., Abdelaziz S., Abdalla R.H. (2018). Anticancer activity and concurrent analysis of ursolic acid, β-sitosterol and lupeol in three different *Hibiscus* species (aerial parts) by validated HPTLC method. Saudi Pharm. J..

[9] Janson B., Prasomthong J., Malakul W., Boonsong T., Tunsophon S. (2021). *Hibiscus sabdariffa* L. calyx extract prevents the adipogenesis of 3T3-L1 adipocytes, and obesity-related insulin resistance in high-fat diet-induced obese rats. Biomed. Pharmacother..

[10] Riaz G., Chopra R. (2018). A review on phytochemistry and therapeutic uses of *Hibiscus sabdariffa* L.. Biomed. Pharmacother..

[11] Kinupp V.F., Lorenzi H.H. (2014). Unconventional Food Plants (PANC) in Brazil: Identification Guide, Nutritional Aspects and Illustrated Recipes.

[12] Ball D.W. (2006). Concentration Scales for Sugar Solutions. J. Chem. Educ..

[13] Chensom S., Okumura H., Mishima T. (2019). Primary Screening of Antioxidant Activity, Total Polyphenol Content, Carotenoid Content, and Nutritional Composition of 13 Edible Flowers from Japan. Prev. Nutr. Food Sci..

[14] Lee Y.-S., Yang W.-K., Kim H.Y., Min B., Caturla N., Jones J., Park Y.-C., Lee Y.-C., Kim S.-H. (2018). Metabolaid^®^ Combination of Lemon Verbena and Hibiscus Flower Extract Prevents High-Fat Diet-Induced Obesity through AMP-Activated Protein Kinase Activation. Nutrients.

[15] Kakkar S., Bais S. (2014). A Review on Protocatechuic Acid and Its Pharmacological Potential. ISRN Pharmacol..

[16] Sim Y.Y., Nyam K.L. (2020). *Hibiscus cannabinus* L. (kenaf) studies: Nutritional composition, phytochemistry, pharmacology, and potential applications. Food Chem..

[17] Liu J.-Z., Zhang C.-C., Fu Y.-J., Cui Q. (2021). Comparative analysis of phytochemical profile, antioxidant and anti-inflammatory activity from *Hibiscus manihot* L. flower. Arab. J. Chem..

[18] Mar J.M., da Silva L.S., Moreira W.P., Biondo M.M., Pontes F.L.D., Campos F.R., Kinupp V.F., Campelo P.H., Sanches E.A., Bezerra J.d.A. (2021). Edible flowers from *Theobroma speciosum*: Aqueous extract rich in antioxidant compounds. Food Chem..

[19] Da-Costa-Rocha I., Bonnlaender B., Sievers H., Pischel I., Heinrich M. (2014). *Hibiscus sabdariffa* L.—A phytochemical and pharmacological review. Food Chem..

[20] Ormazabal P., Scazzocchio B., Varì R., Santangelo C., D’archivio M., Silecchia G., Iacovelli A., Giovannini C., Masella R. (2018). Effect of protocatechuic acid on insulin responsiveness and inflammation in visceral adipose tissue from obese individuals: Possible role for PTP1B. Int. J. Obes..

[21] Izcara S., Perestrelo R., Morante-Zarcero S., Câmara J.S., Sierra I. (2022). High throughput analytical approach based on μQuEChERS combined with UHPLC-PDA for analysis of bioactive secondary metabolites in edible flowers. Food Chem..

[22] Montalvo-González E., Villagrán Z., González-Torres S., Iñiguez-Muñoz L.E., Isiordia-Espinoza M.A., Ruvalcaba-Gómez J.M., Arteaga-Garibay R.I., Acosta J.L., González-Silva N., Anaya-Esparza L.M. (2022). Physiological Effects and Human Health Benefits of *Hibiscus sabdariffa*: A Review of Clinical Trials. Pharmaceuticals.

[23] Loizzo M.R., Pugliese A., Bonesi M., Tenuta M.C., Menichini F., Xiao J., Tundis R. (2015). Edible Flowers: A Rich Source of Phytochemicals with Antioxidant and Hypoglycemic Properties. J. Agric. Food Chem..

[24] Grajeda-Iglesias C., Salas E., Barouh N., Baréa B., Figueroa-Espinoza M.C. (2017). Lipophilization and MS characterization of the main anthocyanins purified from hibiscus flowers. Food Chem..

[25] Zhang P., Li Y., Chong S., Yan S., Yu R., Chen R., Si J., Zhang X. (2022). Identification and quantitative analysis of anthocyanins composition and their stability from different strains of *Hibiscus syriacus* L. flowers. Ind. Crops Prod..

[26] Mar J.M., da Silva L.S., Lira A.C., Kinupp V.F., Yoshida M.I., Moreira W.P., Bruginski E., Campos F.R., Machado M.B., de Souza T.P. (2020). Bioactive compounds-rich powders: Influence of different carriers and drying techniques on the chemical stability of the *Hibiscus acetosella* extract. Powder Technol..

[27] Ngan L., Tan M., Hoang N., Thanh D., Linh N., Hoa T., Nuong N., Hieu T. (2021). Antibacterial activity of *Hibiscus rosa*-*sinensis* L. red flower against antibiotic-resistant strains of *Helicobacter pylori* and identification of the flower constituents. Braz. J. Med. Biol. Res..

[28] Rengarajan S., Melanathuru V., Govindasamy C., Chinnadurai V., Elsadek M.F. (2020). Antioxidant activity of flavonoid compounds isolated from the petals of *Hibiscus rosa* sinensis. J. King Saud Univ.-Sci..

[29] Afify A.E.-M.M.R., Hassan H.M.M. (2016). Free radical scavenging activity of three different flowers—*Hibiscus rosa*-*sinensis*, *Quisqualis indica* and *Senna surattensis*. Asian Pac. J. Trop. Biomed..

[30] Yin S., Cai Z., Chen C., Mei Y., Wei L., Liu S., Zou L., Wu N., Yuan J., Liu X. (2022). Comparative Study on Chemical Constituents of Medicinal and Non-Medicinal Parts of Flos *Abelmoschus manihot*, Based on Metabolite Profiling Coupled with Multivariate Statistical Analysis. Horticulturae.

[31] Kapepula P.M., Ngombe N.K., Tshibangu P.T., Tsumbu C., Franck T., Mouithys-Mickalad A., Mumba D., Tshala-Katumbay D., Serteyn D., Tits M. (2017). Comparison of metabolic profiles and bioactivities of the leaves of three edible Congolese *Hibiscus* species. Nat. Prod. Res..

[32] Lyu J.I., Ryu J., Jin C.H., Kim D.-G., Kim J.M., Seo K.-S., Kim J.-B., Kim S.H., Ahn J.-W., Kang S.-Y. (2020). Phenolic Compounds in Extracts of *Hibiscus acetosella* (Cranberry Hibiscus) and Their Antioxidant and Antibacterial Properties. Molecules.

[33] Parmenter B.H., Croft K.D., Hodgson J.M., Dalgaard F., Bondonno C.P., Lewis J.R., Cassidy A., Scalbert A., Bondonno N.P. (2020). An overview and update on the epidemiology of flavonoid intake and cardiovascular disease risk. Food Funct..

[34] Bondonno N.P., Liu Y.L., Zheng Y., Ivey K., Willett W.C., Stampfer M.J., Rimm E.B., Cassidy A. (2023). Change in habitual intakes of flavonoid-rich foods and mortality in US males and females. BMC Med..

[35] Abdul-Awal S.M., Nazmir S., Nasrin S., Nurunnabi T.R., Uddin S.J. (2016). Evaluation of pharmacological activity of *Hibiscus tiliaceus*. Springerplus.

[36] Μatsumoto T., Imahori D., Achiwa K., Saito Y., Ohta T., Yoshida T., Kojima N., Yamashita M., Nakayama Y., Watanabe T. (2020). Chemical structures and cytotoxic activities of the constituents isolated from *Hibiscus tiliaceus*. Fitoterapia.

[37] Zenebon O., Pascuet N.S., Tiglea P., Adolfo Lutz Institute (2008). Physico-Chemical Methods for Food Analysis.

[38] Molyneux P. (2004). The Use of the Stable Free Radical Diphenylpicryl-Hydrazyl (DPPH) for Estimating Antioxidant Activity. Songklanakarin J. Sci. Technol..

[39] Re R., Pellegrini N., Proteggente A., Pannala A., Yang M., Rice-Evans C. (1999). Antioxidant activity applying an improved ABTS radical cation decolorization assay. Free Radic. Biol. Med..

[40] Velioglu Y.S., Mazza G., Gao L., Oomah B.D. (1998). Antioxidant Activity and Total Phenolics in Selected Fruits, Vegetables, and Grain Products. J. Agric. Food Chem..

[41] Ramos A.S., Mar J.M., da Silva L.S., Acho L.D., da Silva B.J.P., Lima E.S., Campelo P.H., Sanches E.A., Bezerra J.A., Chaves F.C.M. (2019). Pedra-ume caá fruit: An Amazon cherry rich in phenolic compounds with antiglycant and antioxidant properties. Food Res. Int..

[42] Ramos A.S., Souza R.O., Boleti A.P.D.A., Bruginski E.R., Lima E.S., Campos F.R., Machado M.B. (2015). Chemical characterization and antioxidant capacity of the araçá-pera (*Psidium acutangulum*): An exotic Amazon fruit. Food Res. Int..

[43] Lima L.G.B., Montenegro J., de Abreu J.P., Santos M.C.B., Nascimento T.P.D., Santos M.d.S., Ferreira A.G., Cameron L.C., Ferreira M.S.L., Teodoro A.J. (2020). Metabolite Profiling by UPLC-MS^E^, NMR, and Antioxidant Properties of Amazonian Fruits: Mamey Apple (*Mammea americana*), Camapu (*Physalis angulata*), and Uxi (*Endopleura uchi*). Molecules.

[44] Wang H., Provan G.J., Helliwell K. (2003). HPLC determination of catechins in tea leaves and tea extracts using relative response factors. Food Chem..

[45] da Silva U.P., Ferreira B.W., de Sousa B.L., Barreto R.W., Martins F.T., Neto J.H.D.A., Vaz B.G., da Silva R.R., Martins T.V.F., Mendes T.A.D.O. (2022). Synthesis of bis(ylidene) cyclohexanones and their antifungal activity against selected plant pathogenic fungi. Mol. Divers..

[46] Nascimento F.R., Baeta J.V.D.P.B., de França A.A.P., e Oliveira M.A.B.R., Pizziolo V.R., dos Santos A.A., Mendes T.A.D.O., Diaz-Muñoz G., Diaz M.A.N. (2021). Dibenzoylmethane derivative inhibits melanoma cancer in vitro and in vivo through induction of intrinsic and extrinsic apoptotic pathways. Chem. Interact..

